# Investigation of nutritional status, taste-smell functions, and hedonic pleasure of recipients after liver transplantation

**DOI:** 10.3389/fnut.2026.1778028

**Published:** 2026-03-05

**Authors:** Fadime Cinar, Semra Bulbuloglu, Serdar Sarıtaş

**Affiliations:** 1 Division of Surgical Nursing, Nursing Department, Faculty of Health Sciences, Istanbul Nisantasi University, Istanbul, Türkiye; 2 Division of Surgical Nursing, Department of Nursing, Faculty of Health Sciences, Istanbul Aydın University, Istanbul, Türkiye; 3 Department of Midwifery, Faculty of Health Sciences, Istanbul Aydın University, Istanbul, Türkiye; 4 Department of Medical Biology, Faculty of Medicine, Malatya Turgut Ozal University, Malatya, Türkiye

**Keywords:** hedonic pleasure, liver recipients, liver transplantation, nutritional status, smell, taste

## Abstract

**Background:**

Taste and smell disorders have the potential to be associated with malnutrition and a loss of hedonic pleasure in liver transplantation recipients. Information regarding the deterioration of nutritional status in individuals with chronic liver disease is widely known, but changes following liver transplantation need to be investigated.

**Objective:**

This study aimed to investigate the nutritional status, taste and smell functions, and hedonic pleasure of liver transplant recipients.

**Method:**

The data for this descriptive and cross-sectional study were collected by researchers at an organ transplant hospital. The sample group consisted of *n* = 326 liver transplant recipients who met the selection criteria. The data collection tools used in this study were a personal information form, the Controlling Nutritional Status Tool (CONUT), the Complete Mouth Test (CMT), the Connecticut Olfactory Recognition Test (CCCRC), the Food Cravings Questionnaire (FCQ), and the Charlson Comorbidity Index. Information about the data collection tools is provided below. Descriptive tests and parametric tests were used in data analysis.

**Results:**

In this study, 62.9% of liver transplant recipients were moderately malnourished, 62.9% had severe hyposmia, and 74.2% had hypogeusia. There was an inverse correlation between the duration post-transplant and the severity of malnutrition, taste and smell disorders, and a direct correlation with perceived hedonic pleasure (*p* < 0.05). Accordingly, there was a statistically significant negative and weak correlation between FCQ and CONUT mean scores (*r* = −0.154, *p* = 0.005). There was a statistically significant negative and weak correlation between CMT mean scores and CONUT (*r* = −0.142, *p* = 0.011). There was a statistically significant negative and moderate correlation between the CCCRC score average and age (*r* = −0.432, *p* = 0.000) and a statistically significant negative and weak correlation between the CCCRC score average and CONUT (*r* = −0.158, *p* = 0.004).

**Conclusion:**

This study revealed that more than half of liver transplant recipients were moderately malnourished, with a similar proportion having severe hyposmia, and three-quarters had hypogeusia. The severity of malnutrition, along with taste and smell disorders, decreased with increasing post-transplant duration. Smell deficits increased with age. Strategies should be developed to improve malnutrition, taste, and smell disorders in liver transplant recipients, particularly the elderly and those in the early post-transplant period.

## Introduction

Liver transplantation has been the only curative treatment option for end-stage liver disease (1), since the 1980s (2). The estimated 1-year survival rate in adults who have undergone primary liver transplantation has been reported to be over 90% (3). In addition to good surgical techniques and imaging methods, effective immunosuppressive drug regimens and high patient compliance with these regimens enable the prevention of morbidity after liver transplantation and the achievement of high survival rates (4). The liver acts as a barrier between nutrients and microflora in the systemic circulation and the enteral system (5). Through a series of processes occurring in the liver, the body is protected from pathogenic infections and malignant cells, and nutrients are transported to the body via the portal vein (6). Nutritional disorders and malnutrition-related problems have become universal in individuals with end-stage chronic liver disease (7).

With the liver becoming functional after transplantation, many metabolic disorders improve, and changes in body composition are expected in liver transplant recipients who have adopted a good nutrition protocol and lifestyle. Previous studies have reported an increase in fat mass and minimal improvement in muscle mass in sarcopenic recipients after a short period of 12 months (8–10). Muscle loss in chronic liver disease varies by gender; sarcopenia is more pronounced in men because women have lower muscle mass physiologically (11–13). There is insufficient evidence regarding the reasons for the lack of desired increases in muscle mass, especially in the first year after liver transplantation. During this process, taste and smell disorders may be an important determinant of nutritional problems. Olfactory dysfunction in the brain may be caused by a small cytokine storm in the mucosa and olfactory nerve due to a chemical/biological agent or inflammation (14). A previous study reported that the effects of hepatic encephalopathy were still observed in liver transplant recipients even at the end of the 2-year post-transplant period (15).

Neurodegeneration caused by chronic liver disease can impair olfactory structures. Psychiatric disorders, cirrhotic diseases, and intracranial tumors are also associated with olfactory dysfunction (16, 17). From this perspective, liver transplant recipients may be at risk of impaired taste and smell functions for a long period, including the few years following transplantation. This may interfere with hedonic pleasure. Hedonic pleasure is defined as deriving pleasure from food consumption independently of homeostatic eating behavior. Homeostatic hunger arises as a result of decreased blood glucose levels and increased free fatty acid levels, leading to a tendency to turn to food to meet energy requirements and suppress hunger. Deriving pleasure from the act of eating falls under the definition of hedonic eating (18). The relationship between the nutritional status of liver transplant recipients and their taste-smell functions and hedonic pleasure has not been previously investigated in depth. This study aims to monitor the nutritional status of liver transplant recipients and to examine their taste-smell functions and hedonic pleasure levels. The research primary question of this study is whether impaired taste and smell functions in liver transplant recipients have associations on hedonic pleasure and malnutrition differing in the time period after liver transplantation.

## Materials and methods

### Research design and sample

This study aimed to investigate the nutritional status, taste-smell functions, and hedonic pleasure of liver transplant recipients. This study is a descriptive and cross-sectional study. The data collection period was between January 20 and May 15, 2025. Data collection was conducted by researchers at an organ transplant hospital in eastern Turkey. Before this study, ethical committee approval and institutional review board approval were obtained. The sample consisted of liver transplant recipients who met the selection criteria. To calculate the sample size, the G Power 3.1.9.7 software program was used. According to this program, a minimum of 124 participants were required with an effect size of 0.8, a margin of error of 0.05, a confidence interval of 0.95, and a power of 95% to represent the population. The sample for this study consisted of 326 liver transplant recipients. The selection and exclusion criteria for the sample are shown below.

### Inclusion and exclusion criteria

The inclusion criteria for this study were (i) liver transplant recipients who agreed to participate in the study, (ii) having been on an immunosuppressive drug regimen for at least 3 months, (iii) 18 years or older and no communication problems, and having no communication issues, (iv) recipients whose hospital stay following liver transplantation had been completed and only ambulatory stable recipients. The exclusion criteria for this study were (i) recipients whose immunosuppressive treatment had been completed or who were still hospitalized, (ii) recipients who had not completed 3 months post-liver transplantation and were not willing to participate in the study, (iii) patients under the age of 18 with communication or language barriers, and (iv) recipients whose treatment was still ongoing in the hospital.

### Data collection tools

The data collection tools used for liver transplant recipients were the Personal Information Form, Controlling Nutritional Status Tool (CONUT), Complete Mouth Test (CMT), the Connecticut Chemosensory Clinical Research Center (CCCRC), Charlson Comorbidity Index (CCI), and Food Cravings Questionnaire (FCQ). Information about the data collection tools is presented below.

### CONUT

CONUT is used to assess nutritional status and was developed by Ignacio de Ulíbarri et al. (19). CONUT evaluates patients’ nutritional levels based on biochemical findings. It uses albumin, lymphocyte, and cholesterol values as a basis.

The values obtained from the CONUT calculation are scored as follows: serum albumin concentration ≥ 3.5 (g/dL) is scored as 0 points; 3.0–3.49 (g/dL) is scored as 2 points; 2.5–2.9 (g/dL) is scored as 4 points; and < 2.5 (g/dL) is scored as 6 points. If the lymphocyte count is ≥ 1.60 (mm^3^), 0 points are given; if it is 1.20–1.59 (mm^3^), 1 point is given; if it is 0.80–1.19 (mm^3^), 2 points are given; and if it is < 0.8 (mm^3^), 3 points are given. Total cholesterol ≥ 180.00 mg/dL is assigned 0 points; 140.00–179.99 (mg/dL) is assigned 1 point; 100.00–139.99 (mg/dL) is assigned 2 points; and < 100.00 (mg/dL) is assigned 3 points. These scores have been validated (19). The ranges of total scores obtained from CONUT were 0–1 for normal, 2–4 for mild, 5–8 for moderate, and 9–12 for severe malnutrition.

### CMT

The CMT (20) is a taste disorder detection test using taste solutions. The CMT is based on the perception or lack thereof of sweet, salty, bitter, and sour tastes. Sucrose (sweet), sodium chloride (salty), quinine hydrochloride (bitter), and citric acid (sour) are used as stimuli to determine the perception of the four basic tastes. Four separate concentrations were obtained for each taste at mild, moderate, severe, and very severe levels. Liver transplant recipients were asked to sip a total of 16 different liquids, each representing one of the four tastes, in a random order, and then rinse their mouths and spit out the liquid. After each test, recipients were asked to name the taste they perceived. One point was given for correct identification and zero points for incorrect identification. The CMT score range is 0 points for ageusia, 1–4 points for hypogeusia, 5–12 points for normogeusia, and 13–16 points for hypergeusia.

### CCCRC

CCCRC includes both smell detection and identification tests. Detection thresholds are measured using nine separate butanol serial dilutions diluted with deionized water. Each concentration is presented to the patient in a double-blind manner with a water control. The threshold is determined after four consecutive correct responses. If the patient selects water among these responses, the test is repeated at a higher concentration (21, 22). Finally, the detection threshold and identification scores are combined. Veyseller et al. (23) applied the CCCRC to 426 healthy volunteers in our country and stated that the odors used in the test are familiar to the Turkish community. When examining the scores obtained from the CCCRC, the 90–100 point range indicates normal, the 70–80 point range indicates mild hyposmia, the 50–60 point range indicates moderate hyposmia, the 20–40 point range indicates severe hyposmia, and the 0–10 point range indicates anosmia.

### FCQ

FCQ was developed by Cepeda-Benito et al. (24) in 2000. The Turkish validity and reliability study was conducted by Müftüoðlu et al. (25). The scale consists of 9 subscales and 39 items. The Cronbach Alpha internal consistency coefficient for the entire FCQ was found to be 0.97. The reliability coefficients and item counts for the 9 subscales of the Turkish FCQ are as follows: 0.76 and 3 items for the 1st subscale (intention and plan to consume food), 0.81 and 5 items for the 2nd subscale (anticipation of positive reinforcement from eating) is 0.81 with 5 items, the 3rd subscale (anticipation of relief from negative emotions and situations as a result of eating) is 0.84 with 3 items, the 4th subscale (lack of control over eating) is 0.89 with 6 items, the 5th subscale (thoughts and mental preoccupation with food) for 0.93 and 7 items, 6th subscale (experiencing excessive physiological cravings) for 0.78 and 4 items, 7th subscale (experiencing excessive food cravings and emotions during or before eating) for 0.88 and 4 items, 0.85 and 4 items for the 8th subscale (cues that may trigger food cravings), and 0.79 and 3 items for the 9th subscale (guilt from cravings and/or for giving into them). All items in the FCQ are 6-point Likert-type, with responses ranging from 6 = Always, 5 = Most of the time, 4 = Often, 3 = Sometimes, 2 = Rarely, 1 = Never. The FCQ score range is 39 to 234. Accordingly, an increase in FCQ scores indicates an increase in hedonic pleasure (25). In this study, the Cronbach’s Alpha coefficient was found to be 0.89.

### CCI

CCI evaluates 19 conditions and comorbidities and scores them accordingly. The CCI was developed by Charlson et al. (26). The CCI is used to calculate the 1-year mortality rate and categorize diseases. Scores are assigned on a scale of 1, 2, 3, and 6 based on the risk level and scores used to estimate the mortality rate. CCI score accounts for comorbidities and their severity to assign a final score.

### Evaluation of research data

The data from this study were analyzed using Statistical Package for the Social Sciences (SPSS) 27.0, IBM (Armonk, NY). First, the Kolmogorov–Smirnov test was used to determine the presence of normal distribution. Descriptive statistical methods (frequency, percentage, mean, standard deviation, minimum, and maximum values) were used to calculate numbers, percentages, and means. The independent sample *t*-test and one-way analysis of variance (ANOVA) were used to compare independent groups. The *post hoc* Scheffe test was used to determine the source of statistical differences. The Pearson correlation test and multiple linear regression were used to determine the relationship between variables. The numerical results obtained were evaluated at a statistical significance level of *p* < 0.05 and a 95% confidence interval.

### Ethical considerations

Before commencing this study, ethical approval was obtained from the Ethics Committee of Nişantaşı University (Date: January 6, 2025, Decision No: SBETKK2025-01). Subsequently, Institutional Review Board approval was obtained from the Liver Transplantation Institute at Turgut Özal Medical Center (Date: January 15, 2025, No: E-93629378-605-543184). Informed written consent was obtained from each recipient by the Helsinki Declaration.

### Findings

Table 1 shows the personal and health-related characteristics of liver transplant recipients. The sample for this study included *n* = 326 liver transplant recipients. Thirty-six point eight percent of liver transplant recipients were between the ages of 48 and 57, 63.2% were male, and 80.4% had a no comorbid condition. 46.3% of liver transplant recipients were between 6 months and 1 year post-transplant, and all were taking immunosuppressive drugs. When the sense of smell of liver transplant recipients was examined, 28.8% had anosmia, 62.9% had severe hyposmia, and 8.3% had moderate hyposmia. When the taste perception of liver transplant recipients was examined, 17.5% had ageusia, 74.2% had hypogeusia, and 8.3% had normogeusia. The mean age, post-transplant hospital stay, CCI score, and MELD score were 43.29 ± 13.70, 20.48 ± 8.53, 1.34 ± 0.40, and 16.82 ± 1.80, respectively. There were no patients with normal nutrition status, with a prevalence 62% of moderate and 0.9% of severe malnutrition according to the CONUT score.

**TABLE 1:** Personal and health-related characteristics of liver transplant recipients (*n* = 326).

Means	$\bar{x}$ ± SD	Min–max
Age	43.29 ± 13.70	19–76
Post-transplant hospital stay duration	20.48 ± 8.53	9–46
CCI score	1.34 ± 0.40	1–3
MELD score	16.82 ± 1.80	8–19
**Categorical characteristics**	***n***	**%**
Age		
18 to 27 range	49	15
28 to 37 range	70	21.5
38 to 47 range	66	20.2
48 to 57 range	120	36.8
58 to 76 range	21	6.4
Gender		
Female	120	36.8
Male	206	63.2
Comorbidity		
Yes	64	19.6
No	262	80.4
Multiple immunosuppressive drug use		
Yes	326	100
Elapsed time after transplant		
3 to 6 months	64	19.6
6 months to 1 year	151	46.3
1 to 2 years	90	27.6
More than 2 years	21	6.4
Malnutrition (according to CONUT)		
Mild	121	37.1
Moderate	202	62
Severe	3	0.9
Sense of smell		
Anosmia	94	28.8
Severe hyposmia	205	62.9
Moderate hyposmia	27	8.3
Sense of taste		
Ageusia	57	17.5
Hypogeusia	242	74.2
Normogeusia	27	8.3

Table 2 shows the CONUT, CMT, CCCRC, FCQ total, and subscale mean scores of liver transplant recipients. The CONUT mean score was 5.09 ± 1.28 and was in the moderate malnutrition range. In addition, the lowest CONUT score was 2, and the highest was 12. The CMT mean score was 2.54 ± 1.66 and corresponded to hypogeusia. The CCCRC score average was 26.65 ± 14.93, corresponding to severe hyposmia. The FCQ score average was 109.09 ± 15.44. When the scores obtained from the subscales of the FCQ were examined, “intention and plan to consume food,” “anticipation of positive reinforcement from eating,” “anticipation of relief from negative emotions and situations as a result of eating,” “lack of control over eating,” “thoughts and mental preoccupation with food,” “physiological craving,” “excessive food cravings and emotions experienced during or before eating,” “cues that may trigger food cravings,” and “guilt from cravings and/or for giving into them.” 10.11 ± 3.26, 11.98 ± 4.20, 9.45 ± 3.52, 11.34 ± 2.55, 18.74 ± 6.29, 12.58 ± 4.59, 12.51 ± 4.41, 13.93 ± 3.61, and 8.63 ± 8.63, respectively.

**TABLE 2:** CONUT, CMT, CCCRC, FCQ total and subscale mean scores of liver transplant recipients.

Total and subdimensions of scales	Score range	$\bar{x}$ ± SD	Min–max
CONUT	0–12	5.09 ± 1.28	2–12
CMT	0–16	2.54 ± 1.66	0–6
CCCRC	0–100	26.65 ± 14.93	0–60
FCQ	39–234	109.09 ± 15.44	77–147
Intention and plan to consume food	3–18	10.11 ± 3.26	3–17
Anticipation of positive reinforcement from eating	5–30	11.98 ± 4.20	5–21
Anticipation of relief from negative emotions and situations as a result of eating	3–18	9.45 ± 3.52	3–16
Lack of control over eating	6–36	11.34 ± 2.55	6–19
Thoughts and mental preoccupation with food	7–42	18.74 ± 6.29	7–28
Physiological craving	4–24	12.58 ± 4.59	5–22
Excessive food cravings and emotions experienced during or before eating	4–24	12.51 ± 4.41	5–21
Cues that may trigger food cravings	4–24	13.93 ± 3.61	9–23
Guilt from cravings and/or for giving in to them	3–18	8.63 ± 8.63	3–17

Table 3 shows the correlation analysis between variables and scale scores. According to this, there is a weak negative correlation between the FCQ mean score and the length of hospital stay after transplantation (*r* = −0.102, *p* = 0.0027), a positive and moderate correlation between the CCI score (*r* = 0.378, *p* = 0.000), and a negative and weak correlation between the CONUT score average (*r* = −0.154, *p* = 0.005). There was a statistically significant negative and moderate correlation between the CMT score average and age (*r* = −0.359, *p* = 0.000), and a statistically significant negative and weak correlation between the CMT score average and CONUT (*r* = −0.142, *p* = 0.011). There was a statistically significant negative and moderate correlation between the mean CCCRC score and age (*r* = −0.432, *p* = 0.000) and a statistically significant negative and weak correlation between the mean CCCRC score and CONUT (*r* = −0.158, *p* = 0.004). There was a statistically significant positive and weak correlation between the CONUT score mean and age (*r* = 0.244, *p* = 0.000).

**TABLE 3:** Correlation analysis between variables and scale scores.

Correlations		Age	Post-transplant hospital stay duration	MELD score	CCI score	CONUT
FCQ	Pearson correlation	−0.102	−0.122	0.077	0.378	−0.154
	Sig. (2-tailed)	0.067	**0.027** *	0.164	**0.000** **	**0.005** **
	*N*	326	326	326	326	326
CMT	Pearson correlation	−0.359	−0.050	0.086	−0.015	−0.142
	Sig. (2-tailed)	**0.000** **	0.370	0.122	0.793	**0.011** *
	*N*	326	326	326	326	326
CCCRC	Pearson correlation	−0.432	−0.085	0.076	0.027	−0.158
	Sig. (2-tailed)	**0.000** **	0.124	0.170	0.621	**0.004** **
	*N*	326	326	326	326	326
CONUT	Pearson correlation	0.244	0.067	−0.038	−0.025	–
	Sig. (2-tailed)	**0.000** **	0.228	0.494	0.656	
	*N*	326	326	326	326	

*Correlation is significant at the 0.05 level (2-tailed).

**Correlation is significant at the 0.01 level (2-tailed).

Table 4 shows the relationship between gender and the time elapsed after transplantation in liver transplant recipients and the scales. No statistically significant relationship was found between gender and CONUT, CMT, and FCQ scores in liver transplant recipients (*p* > 0.05). Men had higher CCCRC scores than women, and this difference was statistically significant (*p* = 0.046). As the time elapsed after transplantation increased, the CONUT score decreased (*p* = 0.000), while the CMT, CCCRC, and FCQ scores increased (*p* = 0.013, *p* = 0.002, and *p* = 0.007, respectively), and these differences were statistically significant. The data showed patients with more time since transplantation have better CONUT, CMT, CCCRC, and FCQ scores.

**TABLE 4:** Relationship between the time elapsed after transplantation and FCQ, CMT, CCCRC, and CONUT in liver transplant recipients.

Characteristics	Scales ($\bar{x}$ ± SD)			
	CONUT	CMT	CCCRC	FCQ
Gender				
Female	4.98 ± 1.34	2.13 ± 1.91	23.13 ± 17.32	116.22 ± 13.84
Male	5.11 ± 1.27	2.64 ± 1.59	27.47 ± 14.24	107.45 ± 15.34
Test and Sig.	*t* = 0.757, *p* = 0.385	*t* = 2.885, *p* = 0.090	*t* = 4.015, *p* = 0.046*	*t* = 2.272, *p* = 0.097
Time elapsed after transplantation				
Between 3 and 6 months (1)	6 ± 1.02	2.24 ± 1.53	23.33 ± 14.20	90 ± 14.21
Between 6 months and 1 year (2)	5.29 ± 1.24	2.62 ± 1.64	27.93 ± 16.06	107.44 ± 15.78
Between 1 and 2 years (3)	5.09 ± 1.46	2.85 ± 1.81	30.31 ± 14.36	111.71 ± 11.92
More than 2 years (4)	4.15 ± 1.28	2.93 ± 1.80	31 ± 14.60	134.71 ± 15.21
Test and Sig.	*F* = 10.892, *p* = 0.000**	*F* = 3.632, *p* = 0.013*	*F* = 5.226, *p* = 0.002**	*F* = 1.421, *p* = 0.007**
*Post hoc*	1 > 2, 3 > 4	4, 3 > 1, 2	4, 3 > 2 > 1	4 > 2, 3 > 1

*p < 0.05, **p < 0.01.

**FIGURE 1:**
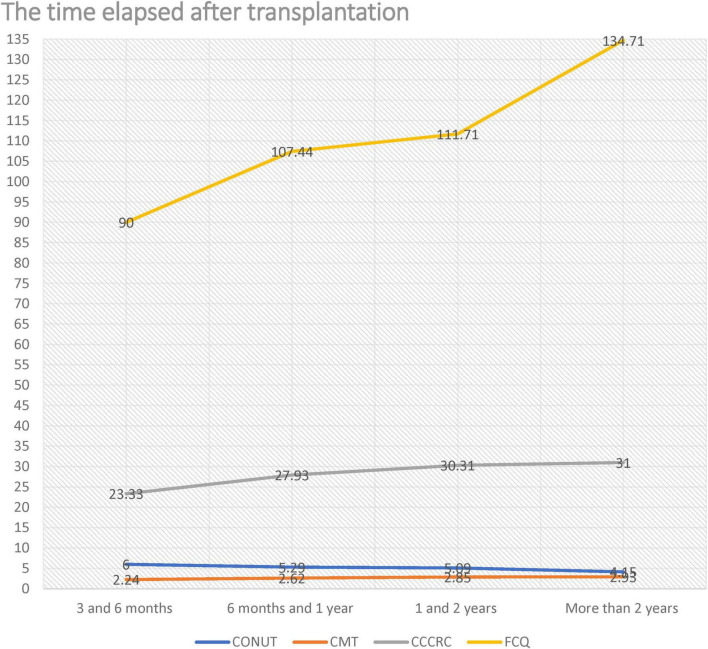
Relationship between the time elapsed after liver transplantation and CONUT, CMT, CCCRC and FCQ.

Figure 1 shows the relationship between the time elapsed after liver transplantation and CONUT, CMT, CCCRC and FCQ. Accordingly, as the time elapsed after liver transplantation increased, malnutrition, taste and smell disorders, and hedonic pleasure improved.

In Table 5, CMT, CCCRC, and FCQ were assigned as independent variables, and CONUT as the dependent variable, and regression analysis was performed. CMT, CCCRC, and FCQ were included in a multiple linear regression model to predict CONUT, and the model was found to be statistically significant (*R* = 0.337, *R*^2^ = 0.114, *F* = 5.603, *p* < 0.001). Accordingly, the model explains 11.4% of the variance in CONUT. When the coefficients are examined, CCCRC negatively and statistically significantly affects CONUT. A 1-unit increase in CCCRC causes a 0.056-unit decrease in CONUT (B = −0.056, Std. Error = 0.034, β = −0.587, *t* = −1.663, *p* = 0.047). A 1-unit increase in FCQ results in a 0.011-unit decrease in CONUT (B = −0.011, Std. Error = 0.005, β = −0.116, *t* = −2.111, *p* = 0.036). In terms of multicollinearity, the tolerance values (0.570 for CCCRC and 0.582 for FCQ) and The Variance Inflation Factor (VIF) values (1.986 for CCCRC and 1967 for FCQ) are acceptable, and the Durbin–Watson coefficient (1.995) indicates the independence of the residuals.

**TABLE 5:** Multiple regression analysis of the relationship between CMT, CCCRC, FCQ, and CONUT.

Independent variable	Dependent variable	Unstandardized B	Coefficients Std. error	Standardized coefficients β	*T*	*p*	*F*	*p*	*R* ^2^
(Constant)	CONUT	6.874	0.559		12.296	< 0.001	5.603	0.000	0.114
CMT		0.371	0.301	0.435	1.236	0.217			
CCCRC		−0.056	0.034	−0.587	−1.663	0.047*			
FCQ		−0.011	0.005	−0.116	−2.111	0.036*			

*p < 0.05, tolerance: 0.570, VIF: 1.031, Durbin–Watson coefficient: 1.995.

## Discussion

In this study, the average age of liver transplant recipients was 43.29, and 93.6% were under the age of 58. The individuals included in this study were faced with chronic liver disease at an age when they should have been productive in terms of work and career, and underwent liver transplantation, which is a major surgical procedure. Thanks to modern surgical techniques and advances in intensive care, survival rates after liver transplantation have been reported to reach 87, 74, and 65% at 1, 5, and 10 years, respectively (27–29). Following liver transplantation, clinicians should aim for “quality survival” rather than mere survival, and holistic care should be provided to this end. The concept of quality survival undoubtedly includes the principle of “supporting patients” functionality. At the individual level, the primary determinant of increased productivity is nutritional status. A person whose protein and energy requirements are not met may not be able to be productive in any way.

According to the results obtained in this study, 37.1% of liver transplant recipients were mildly malnourished, and 62.9% were moderately malnourished, and there was no one who wasn’t malnourished. The CONUT score average corresponded to a moderate malnutrition score. In addition, malnutrition improved as the time after transplantation increased, with severe malnutrition mostly seen between 3 and 6 months after liver transplantation. Malnutrition, recipient age, and MELD score are notable factors among the key determinants of survival after liver transplantation (30, 31), and these factors have the potential to worsen patient prognosis in the postoperative period, increasing morbidity and length of hospital stay (32, 33). In this study, no association was found between MELD score and malnutrition, and the average length of hospital stay was 20.48 days, which was not associated with malnutrition. A previous study reported that malnutrition was associated with the development of hospital-acquired infections (in 8.5% of patients), prolonged intensive care unit stay, and prolonged mechanical ventilation (more than 5 days) after liver transplantation (33). The same study emphasized that sarcopenic patients had longer hospital stays and higher 12-month mortality rates. Studies in the literature indicate that malnutrition in liver transplant recipients often begins in the pre-transplant period and is associated with a wide range of complications, including encephalopathy, peritonitis, and gastrointestinal bleeding (34, 35). Since this study included recipients who had completed at least 3 months post-liver transplantation, we did not examine early clinical outcomes. However, we reported that malnutrition persisted even in recipients who had completed 2 years post-liver transplantation.

In this study, we found that as the length of hospital stay after liver transplantation increased, recipients’ hedonic pleasure decreased. In addition, decreased hedonic pleasure triggers malnutrition (*r* = −0.154, *p* = 0.005). Malnutrition in liver transplant candidates is often caused by increased hypercatabolism, malabsorption, and inadequate calorie intake (36, 37). After liver transplantation, the stress response caused by surgery contributes to the progression of malnutrition and worsens patient prognosis through the development of inflammation, increased sympathetic activity, and the release of catecholamines (37). Malnourished liver transplant recipients are at higher risk for infection, early mortality, and longer morbidity (38, 39). A study involving 373 liver transplant recipients reported a positive correlation between malnutrition and decreased muscle strength, and that both factors independently prolonged the length of stay in the intensive care unit (40). A previous study found that the use of immunosuppressive drugs after liver transplantation was associated with the development of oral lesions (41). It is estimated that lesions in the mouth alter taste perception. Indeed, in this study, we reported that recipients after liver transplantation had more hypogeusia and that taste perception improved over time after transplantation. However, although their compliance levels are unknown, all patients reported using multiple immunosuppressive drugs.

According to the results obtained in this study, the majority of liver transplant recipients had hypogeusia and severe hyposmia. Psychomotor symptoms caused by encephalopathy are frequently encountered in chronic liver disease (42). Taste and smell disorders are common in chronic liver disease and have the potential to continue at varying levels after liver transplantation (43). The literature reports that malnutrition in chronic liver disease patients may contribute to smell loss (44), which is thought to result from the central nervous system effects of liver dysfunction (45). A previous study reported that the olfactory threshold and olfactory discrimination began to deteriorate in patients with early-stage hepatic encephalopathy (40). In the same study, olfactory function was found to be less significant in cirrhotic patients compared to controls, and a relationship was reported between the prevalence of hyposmia and anosmia and the severity of hepatic encephalopathy (40). In this study, taste and smell disorders triggered malnutrition, and hedonic pleasure was highest in recipients who had completed two years and lowest in recipients between three and six months. According to the results of this study, hedonic pleasure decreased as taste and smell disorders increased. In this study, olfactory perception and hedonic pleasure influenced malnutrition by 11.4%. According to our results, a 1-unit increase in good odor perception caused a 0.056-unit decrease in malnutrition (B = −0.056, Std. Error = 0.034, β = −0.587, *t* = −1.663, *p* = 0.047). Similarly, a 1-unit increase in hedonic pleasure caused a 0.011-unit decrease in malnutrition (B = −0.011, Std. Error = 0.005, β = −0.116, *t* = −2.111, *p* = 0.036). Therefore, bad odor perception and weak hedonic pleasure were predictors of malnutrition.

In a study involving 306 liver transplant recipients, it was noted that the rate of patients with persistent liver dysfunction after transplantation was 8.2% and that their mental wellbeing was worse than that of other recipients. The same study reported that even in individuals who had undergone liver transplantation more than two years prior, hepatic encephalopathy did not fully resolve and regressed to stage 1 at most (15). Infection, gastrointestinal bleeding, long-term diuretic use, constipation, psychiatric medications, fluid-volume imbalance, and malnutrition are the leading triggers of hepatic encephalopathy (46). No diagnosis of hepatic encephalopathy was made in this study. However, this connection with malnutrition highlights the importance of initiatives aimed at improving malnutrition in liver transplant recipients. No exemplary studies addressing the hedonic pleasure, taste and smell perception, and malnutrition levels of liver transplant recipients have been found in the literature. From this perspective, the results obtained in this study, which included 326 liver transplant recipients, are of great importance.

### Limitations

This study has several limitations. It was a single-center study. Hepatic encephalopathy screening was not performed in this study, and the immunosuppressive medication compliance of the recipients is unknown. Hepatic encephalopathy and varying immunosuppressive medication compliance may have directly affected taste and smell perception and indirectly affected hedonic pleasure and malnutrition. The present study is the cross-sectional design instead of a longitudinal design, liver transplant recipients were not followed up after data collection. In this study some patient data are lacking such as the indications for liver transplantation, type of underlying liver disease, presence and degree of sarcopenia, body weight, body mass index, type of (immunosuppressive) medication and change in medication over time. Additionally, data collection tools were based on self-reporting by liver transplant recipients, all of which limit the validity of the results.

## Conclusion

This study reported that all liver transplant recipients had varying degrees of malnutrition at 3 months and about 2 years post-transplant. More than half of the liver transplant recipients had moderate malnutrition. Similarly, more than half of liver transplant recipients had severe hyposmia in their sense of smell and hypogeusia in their sense of taste. The factors that reduced the hedonic pleasure felt by liver transplant recipients were a long hospital stay after transplantation and the presence of multiple comorbidities. There was a positive correlation between decreased hedonic pleasure and the severity of malnutrition. In addition, increased taste and smell disorders increased the severity of malnutrition and decreased perceived hedonic pleasure. According to the established model, losses in olfactory sensation and hedonic pleasure were predictors for 11.4% of malnutrition. In this study, with increasing age, there was an increase in taste and smell disorders as well as the severity of malnutrition, while perceived hedonic pleasure decreased. Women’s sense of smell was closer to ageusia than men’s. 2 years after liver transplantation, the malnutrition level of recipients had decreased to a mild level, and taste and smell disorders had improved, reaching the range of hypogeusia and severe hyposmia. In addition, the level of perceived hedonic pleasure had increased.

## Data Availability

The raw data supporting the conclusions of this article will be made available by the authors, without undue reservation.
